# COVID-19-Related Cardiovascular Disease and Practical Considerations for Perioperative Clinicians

**DOI:** 10.1177/1089253220943019

**Published:** 2020-07-24

**Authors:** Neal S. Gerstein, Ranjani Venkataramani, Andrew M. Goumas, Niels N. Chapman, Lev Deriy

**Affiliations:** 1 University of New Mexico, Albuquerque, NM, USA

**Keywords:** COVID-19, SARS-CoV-2, COVID-19 cardiac disease, myocarditis, heart failure

## Abstract

Coronavirus disease 2019 (COVID-19) has a clinical course predominated by acute respiratory failure due to viral pneumonia with possible acute respiratory distress syndrome. However, nearly one third of infected patients, especially those with preexisting cardiovascular (CV) disease, are reported to present with some combination of acute cardiac injury, myocarditis, heart failure, cardiogenic shock, or significant dysrhythmias. In addition, COVID-19 infections are also associated with high rates of thromboembolic and disseminated intravascular coagulation complications. Severe myocarditis and heart failure have both been reported as the initial presenting conditions in COVID-19 infection. This review highlights the important considerations related to the CV manifestations of COVID-19 infections, describes the mechanisms and clinical presentation of CV injury, and provides practical management and therapy suggestions. This narrative review is based primarily on the multiple case series and cohorts from the largest initial COVID-19 outbreak centers (ie, Wuhan, China, and Italy); hence, nearly all presented data and findings are retrospective in nature with the attendant limitations of such reports.

## Introduction

Since its rapid global spread starting in December 2019, the novel β-coronavirus, severe acute respiratory syndrome coronavirus 2 (SARS-CoV-2), which causes coronavirus disease 2019 (COVID-19), has been the focus of the world’s attention.^1^ The clinical course of severe COVID-19 infection is predominated by acute respiratory failure due to viral pneumonia with possible progression to acute respiratory distress syndrome (ARDS). However, nearly one third of infected patients, especially those with preexisting cardiovascular (CV) disease, are reported to present with some combination of acute cardiac injury, myocarditis-associated cardiac dysfunction, or dysrhythmias.^2,3^ Moreover, acute myocarditis and ventricular arrhythmias may be the primary manifestation of severe COVID-19 infection even in the absence of significant respiratory tract involvement.^4^ Chronic CV disease and acute CV disease exacerbations may confound the early diagnosis of COVID-19 due to similar symptomatology (fatigue, cough, and dyspnea) and the mortality from COVID-19 infection is significantly influenced by the degree of myocardial injury.^5^

The following review highlights the important considerations in patients with CV manifestations of COVID-19 infections, describes the mechanisms and clinical presentation of CV injury, and provides potential management and therapy suggestions. It is important to highlight that this narrative review is based primarily on the multiple case series and cohorts from the largest initial COVID-19 outbreak centers (ie, Wuhan, China, and Italy); hence, nearly all presented data and findings are retrospective in nature with the attendant limitations of such reports.

## Perioperative Cardiovascular Issues in COVID-19 Infection

**Figure 1.**
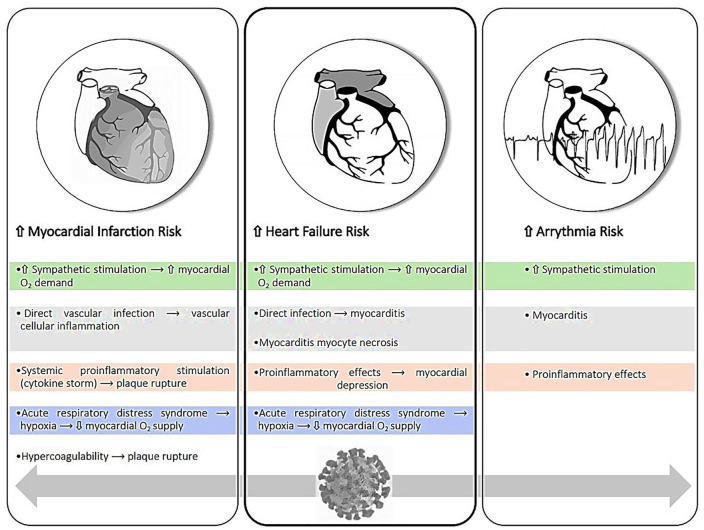
Impact of COVID-19 infection on cardiovascular system and potential pathophysiologic mechanisms.

There is ever-increasing published literature on the CV effects of COVID-19 infection with some of the current knowledge regarding CV sequelae in the current context derived from other recent serious viral respiratory disease outbreaks including SARS-CoV, MERS-CoV (Middle East respiratory syndrome CoV), and H1N1 influenza. Though COVID-19 infection is primarily manifested as a pulmonary disease, the burgeoning literature indicates that COVID-19 infection should be viewed as a systemic disease attacking all organ systems in addition to the respiratory systems.^6^ Though the following separately describes distinct CV entities with attendant complications, there is significant overlap and coexisting pathologies involved in COVID-19-related CV disease (see Figure 1).

### Myocardial Injury

More than 20% of COVID-19 patients have acute myocardial involvement described as “myocardial injury” and is defined by a greater than the 99th percentile of the upper reference limit for high sensitivity troponin-I (hs-TnI).^7,8^ In addition to troponemia, myocardial injury may be variably associated with electrocardiogram and echocardiography abnormalities. The etiology of myocardial injury is not unified and may be due to acute coronary syndrome (ACS; plaque rupture [Type I] or an oxygen supply-demand mismatch [Type II]), a nonischemic process (ie, myocarditis, myopericarditis), or a combination of both. Type I ACS may be related to circulating cytokines released during COVID-19’s severe systemic inflammatory stress response, which may engender atherosclerotic plaque instability and rupture. Type II ACS may be from large increases in myocardial oxygen demand secondary to the infectious process.^9^ Angiotensin-converting enzyme 2 (ACE2) also may play a role in myocardial injury. ACE2 is a membrane-bound aminopeptidase with significant expression in both pulmonary and myocardial tissues. COVID-19 gains entry into human cells via binding ACE2, which in the context of the myocardium may lead to alterations in signaling pathways resulting in myocardial injury.^10^ See section below for further discussion on angiotensin-converting enzyme inhibitors (ACEIs) and angiotensin receptor blockers (ARBs) in the setting of COVID-19.

Troponemia is common in critical illness, particularly in sepsis and ARDS, and shares many pathophysiologic mechanisms of myocardial injury due to COVID-19 infection including altered myocardial supply-demand ratio, systemic inflammation, acute coronary events, and coagulopathy.^10-12^ The risk factors for myocardial injury in critical illness overlap with COVID-19 infection, including but not limited to preexisting CV disease, older age, and diabetes mellitus (DM). Similar to non-COVID-19-related critical illness, the troponin elevation in COVID-19 is strongly associated with severity of the disease and adverse clinical outcomes.^10^ The uniqueness of myocardial injury and troponin leak in COVID-19 infection is in part due to direct viral damage of myocytes with the development of acute viral myocarditis. Viral ribonucleic acid was found in myocardial samples of autopsied hearts in approximately one third of patients who died during the 2003 SARS outbreak.^13^ COVID-19 viral particles have also been found on the endomyocardial biopsy of a patient with COVID-19 infection, supporting the hypothesis of direct myocardial injury by the virus.^7^

Two series from Wuhan, China, provide insight into COVID-19-associated myocardial injury. The first by Shi et al^3^ described 416 hospitalized COVID-19-infected patients of whom 82 (19.7%) met criteria for myocardial injury, and of this subset with cardiac involvement, there was a significantly higher in-hospital mortality rate (51.2% vs 4.5%) with greater degrees of TnI (troponin-I) elevation associated with increased mortality rates.^3^ In the second series by Guo et al,^5^ using troponin-T as the biomarker, in 187 hospitalized COVID-19-infected patients, 52 (27.8%) had a myocardial injury with a mortality rate of 59.6% in those with elevated troponin levels versus 8.9% in those with normal troponin levels. Furthermore, the highest mortality rates were in patients with elevated troponins who had preexisting CV disease (25/36; 69.4%); however, mortality rates were also concerning in those with elevated troponins despite no prior CV disease (6/16; 37.5%). Patients with known CV disease but no troponin elevation also had a relatively poor prognosis with a mortality rate of 13.3%. Guo et al^5^ also reported a direct relationship between levels of C-reactive protein and N-terminal pro-B-type natriuretic peptide levels reflective of myocardial injury, severity of inflammation, and ventricular dysfunction.

Both Guo et al^5^ and Shi et al^3^ found that myocardial injury was more likely to occur in older patients and those with preexisting hypertension (HTN), coronary artery disease (CAD), heart failure, and DM, regardless of whether there was a troponin leak or not. Furthermore, both author groups reported that those with COVID-19 infection and myocardial injury had higher acuity infection with higher rates of ARDS and requirement for mechanical ventilation.

### Arrhythmias

Reports from prior outbreaks along with the current COVID-19 pandemic have been associated with a range of arrhythmogenic issues including palpitations, bradycardia, sinus tachycardia, atrial fibrillation, ventricular fibrillation (VF), ventricular tachycardia (VT), and sudden cardiac death.^14,15^ Wang et al^16^ published on a cohort of 138 hospitalized COVID-19 patients from Wuhan, and arrhythmia was 1 of the 4 reported common complications (16.7% [n = 23]) with a greater prevalence (44.4% vs 6.9%; *P* < .001) in those who were admitted to intensive care unit.^16^ Moreover, cardiac arrhythmias have ranked only second to ARDS in terms of serious complications related to COVID-19 infection.^17^ Initial Chinese reports indicated that ventricular arrhythmias may be the first manifestations of acute myocarditis in COVID-19 infection with VF/VT reported in 5.9% of cohorts.^2,18^

The etiology of these arrhythmogenic issues may stem from metabolic derangements, hypoxia, direct viral myocardial injury, neurohormonal or inflammatory stress, and cardiac structural changes (chamber dilation and dilated cardiomyopathy).^19-21^ In critically ill COVID-19 patients with high rates of arrhythmia (~50% of cases), an acute cardiac injury was only found in half of this cohort (median troponin-I levels in the normal range), indicating that variables outside of direct myocardial damage amplify the arrhythmogenic risks of COVID-19 infection.^21^ Growing evidence indicates that a significant contributor to the arrhythmogenicity of COVID-19 infection involves intense systemic inflammation and that COVID-19-engendered inflammation itself may be a risk factor for long QT-syndrome and torsades de pointes.^21^

The antimalarial agents chloroquine and hydroxychloroquine (HCQ), being tested for use in COVID-19 therapy, should be highlighted in a discussion on arrhythmias. Chloroquine, particularly during long-term use, is known to the lengthen the depolarization duration and refractory period of Purkinje fibers as well as causing atrioventricular node (AVN) dysfunction.^22^ Chloroquine and HCQ are both associated with drug-induced atrial arrhythmias, ventricular arrhythmias, and AVN blockade.^14^ HCQ is known to prolong the QT interval most likely via the blockade of the rapid delayed rectifier channel (I_Kr_), similar to that of chloroquine and quinine, and may induce polymorphic VT and sudden cardiac death.^23,24^ HCQ’s serious adverse effect may be exacerbated by electrolyte perturbations, in the context of other dysrhythmias, or with the use of other QT interval prolonging drugs.^25^ Last, both HCQ and chloroquine inhibit CYP2D6, which may increase β-blocker exposure and risk of bradycardia, PR interval prolongation, and AVN blockade.^1^

### Thromboembolism and Disseminated Intravascular Coagulation

COVID-19-infected patients are at increased risk for thromboembolic risks with up to 71.4% of patients who die from COVID-19 infection meeting the International Society on Thrombosis and Haemostasis criteria (platelet count, prothrombin time, fibrinogen, D-dimer, antithrombin, and protein C activity) for disseminated intravascular coagulation (DIC).^6^ DIC in this context is primarily a prothrombotic version with high rates of venous thromboembolism (VTE), high levels of D-dimer, high fibrinogen, microvascular thrombi in pulmonary vasculature, and high rates of vascular thrombotic events.^26,27^ D-dimer and fibrin/fibrinogen degradation product levels may be especially predictive of COVID-19 disease progression with DIC a feature in the majority of deaths reported in one series; hence, their use in disease management may be warranted.^27,28^ In nearly 90% of a series of hospitalized Chinese COVID-19 patients with pneumonia, there was increased coagulation activity including markedly elevated D-dimer levels with levels greater than 1 µg/mL associated with significant mortality.^29^

Similar to the other CV-related COVID-19 complications, the etiology of increased VTE rates is multifactorial and may be related to a direct viral effect (viral binding to ACE2 endothelial receptor), immobilization, inflammation (causing hypercoagulable state and endothelial dysfunction), underlying CV or CV risk factors (CAD, DM, HTN, and obesity), or preexisting hypercoagulability.^6^ Moreover, the diagnosis of pulmonary thromboembolism may be obfuscated due to overlap in signs and symptoms with primary COVID-19 pulmonary infection. Pulmonary thromboembolism should be considered in the context of sudden oxygenation deterioration, respiratory distress, and hypotension. Moreover, the COVID-19 prothrombotic hypofibrinolytic state leads to widespread alveolar deposition of fibrin and diffuse pulmonary microthromboses, ultimately contributing to the manifestation of an atypical ARDS with preserved lung compliance.^30^

Though not well elucidated, an increased risk for heparin-induced thrombocytopenia (HIT) is possible due to COVID-19-generatred immune dysregulation and increased inflammation associated with significant neutrophil extracellular traps and platelet factor-4 release. Hence, clinicians should be assessing all COVID-19 patients under heparin treatment for indices of HIT by performing the 4T score (thrombocytopenia, timing of platelet count fall, thrombosis or other sequelae, and other causes for thrombocytopenia).^6^

### Heart Failure and Cardiogenic Shock

Similar to the impact of other viral respiratory infections (ie, influenza) associated with both increased rates of heart failure–related admissions and heart failure exacerbations, heart failure is the presenting diagnosis in up to 23% of COVID-19 patients and follows sepsis and ARDS as a common COVID-19 infection complication.^29,31^ In one of the larger reported COVID-19 case series (n = 799) from Wuhan, China, heart failure was a reported complication in 49% of deceased patients with nearly half of these heart failure–complicating deaths having no prior history of HTN or CV disease.^32^ A second series reported heart failure in 52% of those who died as compared with 12% in survivors.^29^

Heart failure and cardiogenic shock etiology in COVID-19 infection is also multifactorial with contributions from the exacerbation of preexisting left ventricular (LV) dysfunction, a new cardiomyopathy (either due to myocarditis or stress cardiomyopathy), hypoxia, hyperadrenergic state, and elevated metabolic demands.^10^ Right-heart failure and associated pulmonary-HTN should also be considered, particularly in the context of severe parenchymal lung disease, ARDS, or possibly pulmonary embolism where right ventricle (RV) dysfunction is common (25% to 50%).^33,34^

Cardiogenic shock may be purely cardiac in origin or may be due to combined cardiac and pulmonary etiologies. In differentiating cardiogenic shock from a mixed etiology, the Berlin criteria may be used with regard to timing of symptom onset, imaging with bilateral pulmonary opacities, and lack of volume overload helps identify patients with ARDS.^35^ BNP, echocardiography, and pulmonary artery catheterization (for filling pressure, mixed venous oxygenation, and cardiac output assessment) all may help guide clinical decision-making, given different management approaches for ARDS and cardiogenic shock. It is crucial to determine whether a concomitant cardiogenic component is present when considering mechanical respiratory and circulatory support with extracorporeal membrane oxygenation (ECMO) or other techniques, as this may lead to changes in cannulation strategies (ie, veno-veno [VV] vs veno-arterial [VA]).^36,37^

Early in the pandemic, Henry and Lippi^37^ reported on the pooled results of 4 Chinese COVID-19-series involving ECMO management. There were 562 COVID-19 patients of which 234 (41.6%) had ARDS; from this group 17/234 (7.2%) received ECMO. Mortality was 94.1% in the ECMO group compared with 70.9% in the standard therapy group. There was no difference in the pooled odds of mortality in those receiving ECMO as compared with the standard therapy (odds ratio = 2.00, 95% confidence interval [CI] = 0.49-8.16) and there was no significant heterogeneity between groups (*I*^2^ = 0%, Cochran’s Q, *P* = .99). In contrast to the Chinese experience, reports from the Extracorporeal Life Support Organization (ELSO) Registry at the time of writing of this document (June 3, 2020) reported 1165 COVID-19 patients (median age = 49 years) were either currently on or had been on ECMO, 91% of these were VV (with the balance VA [4%], VVA [1%], or converted [3%]); they reported that 53% (273/511) of ECMO patients have been discharged alive.^38^ The contrast between the Chinese experience and those reporting to ELSO (94.1% vs 47% mortality) may be related to patient selection differences and variations in institutional management.

### Cardiac Arrest

The only report to date on cardiac arrest is by Shao and colleagues,^39^ who reported on 136 COVID-19 patients with in-hospital cardiac arrest (IHCA) from a single center over a 40-day period between January and February 2020. Of all their reported IHCAs, 87.5% (119/136) were due to a respiratory cause; a cardiac etiology was described in 10/136 and “other” was given for the remaining 7 cases. The initial cardiac rhythm’s in their report were asystole (89.7%), pulseless electrical activity (4.4%), and VF/VT (5.9%); restoration of spontaneous circulation was achieved in 13.2% (18/136), 2.9% survived to 30 days, and there was only 1 patient who was neurologically intact at 30 days. The most frequent comorbidities in this series were HTN (30.2%), DM (19.9%), and CAD (11.0%).^39^ This IHCA report is notable for the high rate of asystole as initial rhythm and poor ROSC (restoration of spontaneous circulation) and 30-day survival rates. Comparatively, IHCA survival-to-discharge in the pre-pandemic era was approximately 20%.^40^

### Echocardiography

There are limited data on the topic of echocardiography in the context of COVID-19 infection. Szekely et al^41^ recently reported on 100 consecutive adult COVID-19 patients (median age = 66 years) who had a complete transthoracic echocardiography examination performed in the first 24 hours of their admission with subsequent examinations performed if their respiratory, cardiac, or hemodynamic condition worsened. One third of the patients (32/100) had a normal initial examination. The most common admission abnormality was RV dilation and RV systolic dysfunction (39%), followed by LV diastolic dysfunction (16%), and LV systolic dysfunction (10%). Initial RV dysfunction was associated with poor clinical condition or an elevated troponin (20%) at admission. In the 20 patients who deteriorated during their admission, the most common echocardiographic finding was RV dilation and dysfunction with a shortened pulmonary acceleration time (10/20; 50% patients) followed by LV systolic and diastolic dysfunction (5 patients). Pulmonary acceleration time (the interval between onset and peak pulmonary flow with shortened times reflecting increased pulmonary vascular resistance) was shorter in older patients, in those with more comorbidities, in those with worse pulmonary disease, and those with higher concentrations of biomarkers (ie, troponin, D-dimer, and brain natriuretic peptide). Though compared with reference values, most patients had increased average mitral E/e′ ratios, a majority of patients (80%) had a mitral E/e′ ratio <14 indicating normal LV filling pressures. Five patients who had RV deterioration were found to have a deep vein thrombosis in the femoral vein system. This report demonstrates that in COVID-19 infection, RV dilatation and systolic dysfunction with a shortened acceleration time are common findings especially in the context of clinical deterioration while LV diastolic dysfunction is more common than isolated LV systolic dysfunction. Moreover, these findings also suggest that another mechanism for troponemia and myocardial injury in COVID-19 may be primarily related to RV dysfunction due to a combination of pulmonary parenchymal disease and pulmonary vascular disease (ie, thromboembolism, microvascular thrombosis, and vasculitis).^41^

## Management Considerations and Therapies in COVID-19 Infection-Related CV disease

See Table 1 for COVID-19 CV sequelae and management recommendations.

**Table 1.** COVID-19 Cardiovascular Sequelae and Management Recommendations.

	Key issues	Management suggestions
Arrhythmias^2,14,21,22,42,43^	• Bradycardia • Sinus tachycardia • Atrial fibrillation • VF/VT • Sudden cardiac death • HCQ-associated QT prolongation • HCQ and chloroquine-induced AV node/Purkinje fiber dysfunction	• Consider early/preoperative placement of defibrillator pads especially if significant myocarditis or myocardial dysfunction • Chloroquine HCQ require ECG monitoring for prolonged QTc, bradycardia, PR interval prolongation, and AVN block • Maintain potassium >4 mEq/L • Maintain magnesium to >2 mg/dL • Possible anti-IL6 agents (ie, tocilizumab)
Myocardial injury^3,5,7,9^	• Defined as troponin leak >99th percentile reference range • May range from mild troponin leak to fulminant severe necrotizing myocarditis • ACS: type I, II, or both • Associated with higher rates of mechanical ventilation and mortality	• Standard ACS work-up/management • Echocardiography (ideally transthoracic) to assist in establishing possible etiology or mechanism of injury
Thromboembolic complications^6,26,27,44,45^	• High risk for VTE and PE • DIC with prothrombotic predominance ○ Elevated D-dimer ○ Moderate-severe thrombocytopenia ○ Low or high fibrinogen depending on presence of DIC ○ Low anti-thrombin • Central line thrombosis •Vascular thrombotic events (ie, CVA, limb ischemia) • HIT in patients receiving heparin products	• Pharmacological thromboprophylaxis to all immobilized and severely ill patients with COVID-19 • Consider PE in any patient with sudden change in oxygenation, BP, or new SOB • In non-bleeding patient, abnormal coagulation results do not require empiric correction • Avoid procoagulants and anti-fibrinolytic agents • Consider anticoagulation/thrombolysis in thrombotic type-DIC • Off-label use of tPA in atypical ARDS
Heart failure and cardiogenic shock^29,32-34,61^	• Presenting diagnosis in up to 23% COVID-19 infection Etiology: ○ Preexisting ventricular dysfunction ○ New cardiomyopathy (myocarditis or stress cardiomyopathy) ○ Hypoxia ○ Hyperadrenergic state ○ Elevated metabolic demands • ~50% of all deaths have associated heart failure complication • Right heart failure and associated pulmonary-HTN should be also considered, particularly in the context of ARDS	• Echocardiography (ideally transthoracic) to assist in establishing possible etiology • Determine whether a concomitant cardiogenic component is present when considering mechanical respiratory and/or circulatory support (ie, Impella, ECMO)
Cardiac arrest^39,47,62,63^	• Primarily respiratory in etiology • For IHCA, low ROSC rate and 30-day survival • Chest compressions cumbersome in PPE gear • CPR is an aerosolizing procedure	• Frequently change individual assigned to compressions • Consider early use of mechanical compression device (ie LUCAS) • Consider VA-ECMO • Have DNR/AND discussion with all hospitalized patients as early as possible

Abbreviations: ACS, acute coronary syndrome; ARDS, acute respiratory distress syndrome; AV, atrioventricular; AVN, atrioventricular node; BP, blood pressure; COVID-19, coronavirus disease 2019; CPR, cardiopulmonary resuscitation; CVA, cerebrovascular accident; DIC, disseminated intravascular coagulation; DNR/AND, do not resuscitate/allow natural death; ECG, electrocardiogram; ECMO, extracorporeal membrane oxygenation; HCQ, hydroxychloroquine; HIT, heparin-induced thrombocytopenia; HTN, hypertension; IHCA, in-hospital cardiac arrest; IL-6, interleukin-6; PE, pulmonary embolism; PPE, personal protection equipment; QTc, corrected QT interval; ROSC, restoration of spontaneous circulation; SOB, shortness of breath; tPA, tissue plasminogen activator; VA-ECMO, venoarterial-ECMO; VF, ventricular fibrillation; VT, ventricular tachycardia; VTE, venous thromboembolism.

### Arrhythmias

First, a heightened preparedness for the management of malignant arrhythmias should be considered in all COVID-19 patients, which may involve preemptive placement of defibrillator pads and insuring easy access to a defibrillator. As severe inflammation may be a driving factor in COVID-19 arrhythmogenicity, attenuating the systemic inflammatory response may affect outcomes.^21^ IL-6 (interleukin-6), a cytokine known to induce cardiac sympathetic system hyperactivation and prolong ventricular myocyte action potentials, is blocked by the monoclonal-antibody tocilizumab, which is currently under investigation in COVID-19 cases.^18^ In limited trials, tocilizumab has demonstrated efficacy in reducing QT intervals in rheumatoid arthritis patients who have higher rates of sudden cardiac death and has shown promise in terms of mitigating myocardial injury related to the inflammatory response.^21,42^

When using chloroquine or HCQ therapies, electrocardiogram monitoring is needed for arrhythmia detection, QT prolongation, torsades de pointe, or atrioventricular blockade. Dose reduction or discontinuation should be considered with QTc (corrected QT interval) > 500 ms or if there is an increase in QTc > 60 ms. In the setting of serious illness, hypokalemia and hypomagnesemia are common findings. To minimize the arrhythmia risk, potassium correction to >4 mEq/L and magnesium to >2mg/dL are recommended.^43^ Additional caution is needed if other QTc-prolonging drugs are used (ie, azithromycin and antiarrhythmics).^1,43^

### Thromboembolism

Because of the high risk for VTE in COVID-19+ patients, early implementation and the prolonged use of low-molecular weight heparin pharmacologic thromboprophylaxis is recommended.^6^ A high index of suspicion for thromboembolic disease including pulmonary embolism needs to be maintained because of the clinical overlap with COVID-19 pulmonary infection. Due to potential drug-drug interactions with concomitant antiviral (ie, ritonavir) and antibacterial (ie, azithromycin) therapies, low-molecular-weight heparins (LMWH) or unfractionated heparin are preferred therapies over direct oral anticoagulants.^6^ Though LMWHs have simplified dosing regimens and a lower risk for HIT as compared with unfractionated heparin, LMWHs are primarily cleared by the kidney and require dose-adjustments in the context of renal dysfunction. Moreover, clinicians should follow the 4T-scoring system to monitor for HIT.

There are 2 published series describing the off-label use of intravenous tissue plasminogen activator (tPA) to treat suspected pulmonary microvascular thrombosis and the associated atypical ARDS. Wang and colleagues^26^ used tPA and heparin in 3 COVID-19-infected mechanically ventilated patients with ARDS and elevated D-dimer levels (>20-50 000 ng/mL) and hyperfibrinogenemia (375-939 mg/dL). All the 3 patients demonstrated improvement in their oxygenation after initiation of tPA therapy. Barrett and colleagues^44^ used tPA and heparin in 5 COVID-19 infected patients with refractory respiratory failure and thrombotic coagulopathy. The authors reported that these 3 patients had sustained improvements after fibrinolytic therapy (including 1 patient extubated 7 days post-tPA), while the other 2 had non-sustained improvements (1 patient remained mechanically ventilated and 1 patient died from multi-organ failure).^44^ Though not yet reported in the context of COVID-19 lung disease, nebulized tPA is an attractive option for selective pulmonary fibrinolysis with fewer systemic bleeding risks.^30^ Further investigation with controlled studies and larger patient groups are needed in order to determine the efficacy and safety of anticoagulation and fibrinolytic therapies use in this context.

Consideration should also be given to minimizing or avoiding the use of antifibrinolytic agents or procoagulants (recombinant activated factor VIIa, prothrombin complex concentrates) in COVID-19-infected in order to mitigate any thromboembolic risks. Moreover, antifibrinolytics should be avoided in DIC because of the need for endogenous fibrinolysis to break down disseminated thrombi.^45^

### Cardiac Arrest and ECMO

Perioperative clinicians should expect the inevitable delays in initiating cardiopulmonary resuscitation for the IHCA COVID-19 patient because of the need to don personal protective equipment (PPE). Even in the context of emergency situations (ie, cardiac arrest), it is imperative that clinicians properly and safely don all needed PPE prior to initiating care; clinician safety should be paramount. Moreover, as described by Shao et al,^39^ cumbersome PPE use makes high-quality cardiopulmonary resuscitation challenging and recommends frequently changing the person assigned to compressions along with early consideration given to a mechanical compression device (ie, LUCAS Chest Compression System), which also allows for fewer potential exposed health care workers.

ECMO use has been reported in a number of the published reports; however, there are limited details or outcome data included in these reports.^37,46^ Interim guidelines have been released by ELSO that aid in the provision of ECMO services to patients with refractory hypoxemia despite optimal mechanical ventilation.^47^ See Table 2 regarding considerations for VV- and VA-ECMO. When considering potential VA- or V-VA ECMO, it should be noted that most patients requiring inotropes pre-VV-ECMO stabilize hemodynamically once the RV strain is relieved by treating hypoxemia and reducing hypoxic pulmonary vasoconstriction on ECMO initiation. It is imperative that cannulation teams be limited to personnel needed to perform and assist with cannulation with the recommendation to have no more than 5 individuals with contact and airborne precautions. Dedicated ECMO cannulation carts with an assistant outside the patient’s intensive care unit room should be established prior to cannulation.^47^ For VV-ECMO, the use of dual cannulas with either the jugulo-femoral or femoro-femoral cannulation strategies are preferable to single dual-lumen internal jugular cannulas due to the latter requiring more sophisticated and frequent re-positioning.

**Table 2.** Considerations for Venovenous and Venoarterial Extracorporeal Membrane Oxygenation in Adult COVID-19 Infection.^47^

Indications/comments		VV- or VA-EMCO contraindications
VV-ECMO	VA-ECMO	
• Same as per typical ELSO and other existing guidelines.^48,49^ • If having exhausted traditional ARDS/lung protective ventilation strategies and therapies (prone positioning, paralysis/high PEEP, recruitment maneuvers, and inhaled pulmonary vasodilators) with: ○ P/F < 60 mm Hg for >6 hours ○ P/F < 50 mm Hg for >3 hours ○ pH < 7.20 + PaCO_2_ > 80 mm Hg for >6 hours • P/F > 150 mm Hg + pH < 7.20 + PaCO_2_ > 80 mm Hg • Single-organ failure either no or minor comorbidities	• Same as per typical ELSO and other existing guidelines.^47,50^ • Consider initiating in refractory cardiogenic shock: ○ Signs of tissue hypoxia ○ SBP < 90 mm Hg ○ CI < 2.2 L/min/m^2^ while receiving vasoactive drug therapy • V-VA configuration may be needed for combined ARDS and cardiogenic shock	• Severe or multisystem disease including multi-organ failure • Immunocompromised (relative) • Terminal disease states including CNS conditions and disseminated malignancy • Intracranial hemorrhage (recent/worsening) • Mechanical ventilation for >14 days before ECMO consideration • Age (institution specific) >65 to 70 years • Severe congenital heart disease • Chronic lung disease • Uncontrolled bleeding, severe risk of bleeding, contraindication to anticoagulation • *NB*: AKI is not a contraindication

Abbreviations: AKI, acute kidney injury; ARDS, acute respiratory distress syndrome; CI, cardiac index; CNS, central nervous system; ECMO, extracorporeal membrane oxygenation; ELSO, extracorporeal life support organization; NB, nota bene; PEEP, positive end-expiratory pressure; P/F, PaO_2_/FiO_2_ ratio; SBP, systolic blood pressure; VA, venoarterial; VV, venovenous.

With ever-increasing evidence of significant hypercoagulability, COVID-19 patients may require more frequent ECMO circuit exchanges. ELSO guidelines highlight the importance of having a primed circuit at all times, avoidance of lower ECMO flow rates (ie, <2 L/min), in addition to targeting a higher end of normal values for anticoagulation. A higher dose of heparin at initiation of ECMO (ie, 7500 units vs 5000 U intravenous) and patients with known hypercoagulable status may benefit from the addition of antiplatelet agents (such as aspirin, clopidogrel, prasugrel, and ticagrelor). In addition to increased clotting events, there seems to be an increased incidence of antithrombin-III deficiency leading to an inability to therapeutically anticoagulate with heparin. A number of centers have switched to bivalirudin as their anticoagulant of choice while on these circuits.^47^

### Angiotensin-Converting Enzyme Inhibitors/Angiotensin Receptor Blockers

There is controversy and lack of substantial data surrounding the use of ACEIs and ARBs in the setting of COVID-19. This controversy is centered on experimental data that support renin-angiotensin-aldosterone system blockade stimulates ACE2 expression and/or activity.^51,52^ ACE2 is a type I integral membrane protein highly expressed in multiple organs (heart, kidney, brain, gut, blood vessels, and lung alveolar cells), providing the main entry site for the virus into human hosts via the SPIKE protein expressed on the SARs-CoV-2.^53^ This enzyme has a high affinity for angiotensin II (Ang II), which it converts to angiotensin 1-7 (Ang-1-7), an antagonist to Ang II, by promoting anti-inflammatory and antifibrotic effects, and the reduction of blood pressure through stimulation of smooth muscle release of nitric oxide.^52,54^ These effects are protective for multiple organ systems in the setting of diabetes, CV disease, and ARDS.^55,56^ This increased expression of ACE2 raises the concern for increased viral infection susceptibility, though the protective effects of ACE2 cannot be discounted. Complications of increased cardiac ACE2 include the development of fulminant myocarditis with increased COVID-19 viral load.^2,57^ It has also been noted there are increased serum levels of Ang II in COVID-19 patients, indicating potential down regulation of ACE2 as seen in SARS-CoV contributing to acute lung injury.^57^ As illustrated by South et al,^52^ there is paucity of data exploring the impact of ACEIs and ARBs on the ACE2-Ang-1-7-Mas receptor axis. Without experimental and clinical data, the true role of ACEI and ARB therapy on COVID-19 infection or progression remains unclear. Given the overall lack of data, a joint statement by the Heart Failure Society of America, American College of Cardiology, and American Heart Association recommends that these medications can be continued in patients with COVID-19 without interruption in compliance with available clinical guidelines and are appropriate in the perioperative setting.^58^

### Transesophageal Echocardiography

Last, consideration should be given to transesophageal echocardiography (TEE), which similar to upper endoscopy, is considered an aerosolizing procedure with the attendant risks of amplified viral transmission. When feasible, transthoracic echocardiography should supplant the use of TEE and if TEE is deemed necessary, the threshold for TEE use should be high and with a clear indication per American Society of Echocardiography (ASE) guidelines.^59^ The ASE published a position article on TEE during the COVID-19 pandemic, which recommends that TEE examinations be “postponed or canceled if they are unlikely to change clinical care, or if an alternative imaging modality can provide the necessary information.”^60^ The ASE writing members also state that TEE should be avoided in non-intubated patients and may be necessary in “urgent or emergent cardiac surgery, patients with cardiac comorbidities undergoing emergent non-cardiac surgery, or hemodynamic instability due to undifferentiated shock in the perioperative period.”^60^ When TEE is required, appropriate PPE should be utilized and the ultrasound system should be as fully covered as possible with a transparent drape especially over frequently touched or difficult to clean surfaces. The ASE document also suggests dedicating one clinician for probe manipulation and a second for instrument adjustment.

## Conclusions

This narrative review, based on the most current COVID-19-related literature, highlights the most significant CV considerations for the perioperative physician. A high percentage of COVID-19-infected patients may initially present with cardiogenic shock that may be related to some combination of myocarditis, ACS, or arrhythmia. It is recommended that all COVID-19 patients be monitored for arrhythmias, as COVID-19-engendered inflammation is a risk factor for malignant arrhythmias along with QT prolongation related to certain therapies. Myocardial injury may be a consequence of decreases in ACE2 due to infection and impairing the protective anti-inflammatory effects that result in ARDS as seen in other SARS-CoV pathology. Thromboembolic risks including VTE are markedly increased in the COVID-19 patient, this effect may be multifactorial due to underlying comorbidities, immobilization, and inflammation with VTE diagnosis made challenging in the already highly pulmonary compromised patient. Based on limited data, initial COVID-19 infection is associated with higher rates of RV systolic dysfunction as compared with the LV, which may be reflective of the underlying pulmonary parenchymal and vascular pathologies. While we await new clinical trials for efficacious therapeutics, vaccines, and research elucidating the underlying mechanisms of CV disease in COVID-19 patients, it is imperative that perioperative physicians remain informed on current management recommendations and future treatment options.
